# Mutations in *Plasmodium falciparum* K13 propeller gene from Bangladesh (2009–2013)

**DOI:** 10.1186/1475-2875-13-431

**Published:** 2014-11-18

**Authors:** Abu Naser Mohon, Mohammad Shafiul Alam, Abebe Genetu Bayih, Asongna Folefoc, Dea Shahinas, Rashidul Haque, Dylan R Pillai

**Affiliations:** Department of Microbiology, Immunology and Infectious Diseases, Faculty of Medicine, University of Calgary, Diagnostic and Scientific Centre, Room 1 W-416, 9-3535 Research Road NW, Calgary, AB T2L 2 K8 Canada; Department of Pathology and Laboratory Medicine, Faculty of Medicine, University of Calgary, Calgary, Canada; Parasitology Research Group, International Centre for Diarrhoeal Disease Research, Dhaka, Bangladesh; Department of Laboratory Medicine and Pathobiology, University of Toronto, Toronto, Canada

## Abstract

**Background:**

Bangladesh is a malaria hypo-endemic country sharing borders with India and Myanmar. Artemisinin combination therapy (ACT) remains successful in Bangladesh. An increase of artemisinin-resistant malaria parasites on the Thai-Cambodia and Thai-Myanmar borders is worrisome. K13 propeller gene (PF3D7_1343700 or PF13_0238) mutations have been linked to both *in vitro* artemisinin resistance and *in vivo* slow parasite clearance rates. This group undertook to evaluate if mutations seen in Cambodia have emerged in Bangladesh where ACT use is now standard for a decade.

**Methods:**

Samples were obtained from *Plasmodium falciparum*-infected malaria patients from Upazila health complexes (UHC) between 2009 and 2013 in seven endemic districts of Bangladesh. These districts included Khagrachari (Matiranga UHC), Rangamati (Rajasthali UHC), Cox’s Bazar (Ramu and Ukhia UHC), Bandarban (Lama UHC), Mymensingh (Haluaghat UHC), Netrokona (Durgapur and Kalmakanda UHC), and Moulvibazar (Sreemangal and Kamalganj UHC).

**Results:**

Out of 296 microscopically positive *P. falciparum* samples, 271 (91.6%) were confirmed as mono-infections by both real-time PCR and nested PCR. The K13 propeller gene from 253 (93.4%) samples was sequenced bi-directionally. One non-synonymous mutation (A578S) was found in Bangladeshi clinical isolates. The A578S mutation was confirmed and lies adjacent to the C580Y mutation, the major mutation causing delayed parasite clearance in Cambodia. Based on computational modeling A578S should have a significant effect on tertiary structure of the protein.

**Conclusion:**

The data suggest that *P. falciparum* in Bangladesh remains free of the C580Y mutation linked to delayed parasite clearance. However, the mutation A578S is present and based on structural analysis could affect K13 gene function. Further *in vivo* clinical studies are required to validate the effect of this mutation.

## Background

The term ‘artemisinin resistance’ in *Plasmodium falciparum* has not been precisely defined. The clinical interpretation is a relatively slow parasite clearance rate in patients receiving artemisinin or artemisinin combination therapy (ACT) [1]. In low malaria transmission areas, parasite clearance studies require screening of thousands of febrile individuals to enroll a few patients. Therefore, these studies can be logistically and financially difficult and inconvenient for patients [2]. From a biological perspective, *in vitro* studies have sought a suitable molecular marker to identify artemisinin resistance in *P. falciparum* parasites. Mutation and variable expression of several genes such as PfMDR and PfATPase6 have been suggested but not proven [3]. Recently, the K13 propeller gene PF3D7_1343700 (PF13_0238) has been linked to *in vitro* artemisinin resistance and *in vivo* slow parasite clearance rate and therefore proposed as marker of artemisinin resistance. K13 propeller has been predicted to consist of three domains 225 amino acid in length: i) *Plasmodium*-specific and well conserved N-terminal domain; ii) a BTB/POZ domain; and, iii) a six-blade C-terminal propeller domain formed of canonical kelch motifs. Kelch motif containing proteins are evolutionary conserved across different species and grouped into KLHL type proteins. The human host contains 42 of these KLHL-type proteins [4]. KLHL19, also known as KEAP1 protein, has maximum homology with *Plasmodium* K13 protein [5]. Human KEAP1 protein is a negative regulator of the inducible nuclear erythroid 2-related factor 2 (Nrf2)-dependent cytoprotective response, sequestering Nrf2 in the cytoplasm under steady state [6, 7]. The transcription factor Nrf2 binds to the antioxidant response element (ARE) present in promoters of genes involved in phase II detoxification and oxidative stress responses. Nrf2 forms a heterodimer to activate Maf transcription factor protein that binds to the ARE and activates transcription through the Maf recognition element (MARE). KEAP1 is involved Nrf-2 degradation by targeting it for ubiquitination through the cullin 3 ligase complex. Therefore, it is assumed that the K13 propeller performs a similar function in the *P. falciparum* and mutation in the gene impairs anti-oxidant/cytoprotective function. However, no orthologue of Nrf2 has been identified in *Plasmodium* parasite genome [5].

Bangladesh is a malaria hypo-endemic country, sharing its border with India and Myanmar. Bangladesh has made great strides in malaria control with a 65% decrease in total malaria cases and 91% reduction in malaria-associated mortality from 2008 to 2012. The number of national cases ranged between 26, 891 and 63, 873 between 2009 and 2013 according to the National Malaria Control Programme. High coverage and increased use of insecticide-treated nets, use of rapid diagnostic tests for case detection at the community level, anti-malarial treatments with ACT, and a high number of community health workers and health facilities have contributed significantly to this achievement [8]. Ministry of Health and Family Welfare (MoHFW) has introduced artemether-lumefantrine (Coartem©) as ACT to treat uncomplicated falciparum malaria since 2004 [9] and the process has accelerated with impetus from the Global Fund in 2007 [8]. Artemisinins are considered as the last line of defense against *P. falciparum*-associated malaria. Since countrywide implementation from 2004, ACT remains quite successful in Bangladesh [10–12]. No day 3 positivity data is available at this time to objectively gauge ACT efficacy. However, the increase of heritable artemisinin-resistant malaria parasite in the Thai-Cambodia border [13], and subsequently in the Thai-Myanmar [14] border to southern Myanmar [15], is worrisome for the Bangladesh-India-Myanmar tri-country border area. Exact population movement in this border area is not defined because huge illegal movements are involved. Rohinga communities of Myanmar have already formed several refugee camps in Bangladesh and still their number is increasing due to civil war in Myanmar. Both transport of resistance via migration and *de novo* emergence of artemisinin resistance are possible. Previously, chloroquine and sulphadoxine/pyrimethamine-resistant parasites emerged in Thai-Cambodia border, consequently spreading to sub-Saharan Africa across the Asia [16, 17]. Hence, monitoring of artemisinin resistance in Bangladesh is an important global health issue. The current study aimed to identify the presence or absence of mutations in the K13 propeller gene of Bangladeshi *P. falciparum* clinical isolates in conjunction with structural analysis to assess the functional implications of mutations present.

## Methods

### Sample collection

*Plasmodium falciparum-*infected malaria patients from corresponding Upazila health complex (UHC) of seven endemic districts of Bangladesh were included in this study. These districts include Khagrachari (Matiranga UHC), Rangamati (Rajasthali UHC), Cox’s Bazar (Ramu and Ukhia UHC), Bandarban (Lama UHC), Mymensingh (Haluaghat UHC), Netrokona (Durgapur and Kalmakanda UHC), and Moulvibazar (Sreemangal and Kamalgonj UHC). Informed consents were obtained from the patients or their legal guardians in the case of children and assent from children aged 11–17 years. A total of 296 *P. falciparum* positive blood samples confirmed by field and laboratory microscopy in between May 2009 and 2013 was collected for this study. Consent was also taken for future use of blood samples. The study was approved by the institutional Ethics Review Committee (ERC) of International Centre for Diarrhoeal Disease Research, Bangladesh **(**icddr,b).

### Real time PCR and nested PCR

DNA was extracted by Qiagen Blood mini kit (Carlsbad, CA) from the collected blood specimens. Microscopy results were confirmed by both real time PCR and nested PCR [18, 19]. If any discrepancies were found by different diagnostic methods, those samples were excluded from the study.

### K13 propeller gene amplification and sequencing

The K13 propeller gene was amplified by the nested PCR method described by Ariey *et al.* [5] with some modifications. The first step of the nested PCR remained unaltered while for the second round of PCR, annealing temperature was adjusted to 65.5°C and annealing time was reduced to 45 sec. Platinum Taq Polymerase (Life Technologies, Ontario, Canada) was used for all of the PCR reactions according to its recommended settings. Amplified products were purified by Qiaquick PCR purification kit (Carlsbad, CA) and then sequenced by Applied Biosystems 3730XL 96 capillary sequencer. ClustalW software [20] was used to align all of the sequences under Unix interface. The sequences in the multiple sequence alignment was edited manually by Jalview software [21] to remove the gaps. ClustalX colour scheme in the Jalview helped to identify the mismatched bases. The sequence of K13 propeller gene was obtained from Genbank (ID: AL844509.2) and used it as reference sequence to locate the point mutations in the clinical isolates.

### Protein structure prediction

Amino acid sequences of the wild type and mutated proteins were used to predict the structure of the protein and effect of mutations found in Bangladeshi clinical isolates. Phyre2 online protein structure prediction tool [22] was used for modelling. The chosen template (PDB ID: 2WOZ) resulted in a model with a confidence score of 100% based on homology assessment and model prediction quality.

## Results

**Figure 1:**
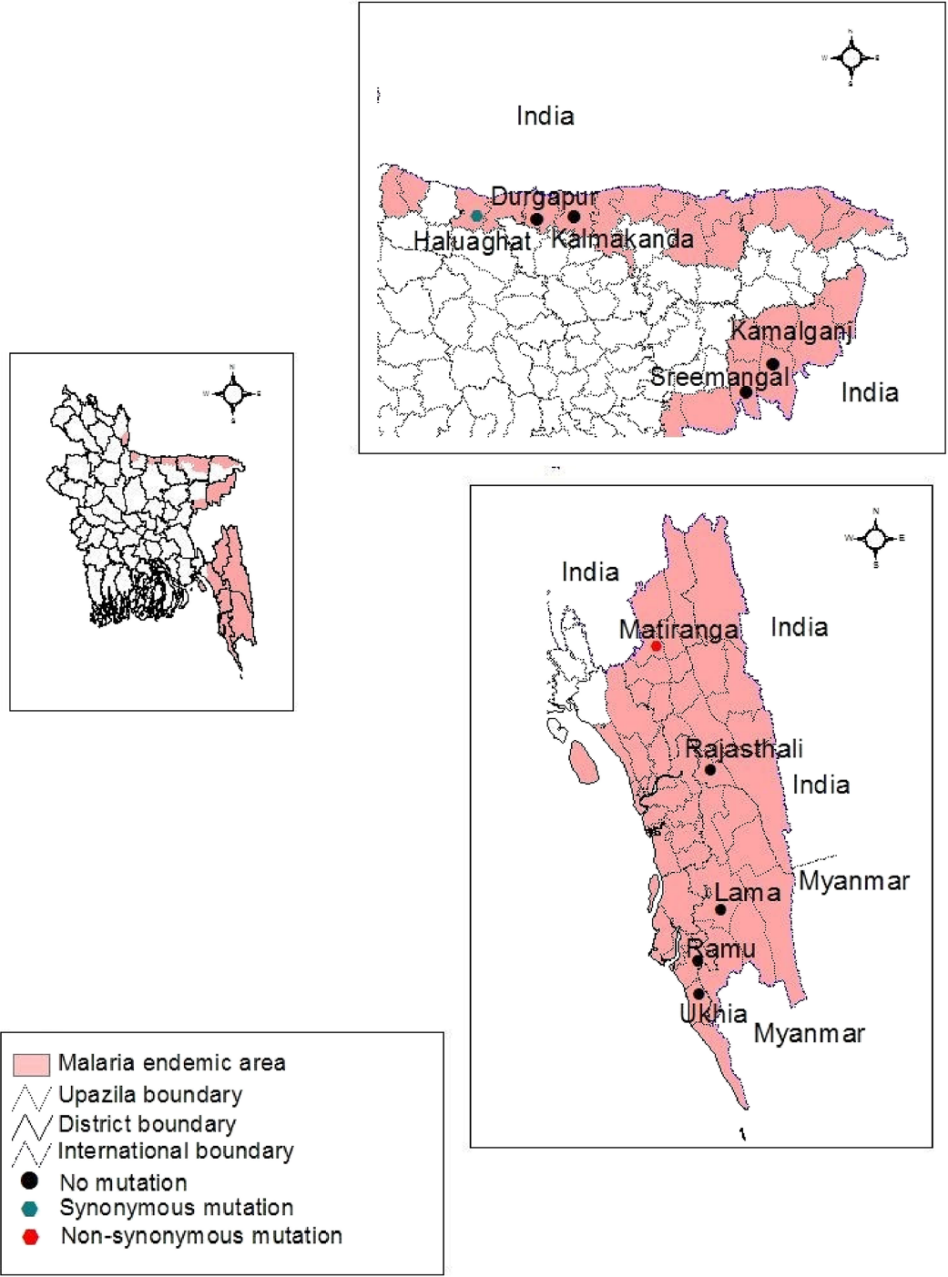
**Geographical distribution of** ***Plasmodium falciparum*** **K13 gene mutations in this study.**

Out of 296 microscopically confirmed *P. falciparum* samples, 271 (91.56%) were confirmed by both real time PCR and nested PCR to have pure *P. falciparum* infection. Other (n = 25) specimens were excluded from the study. With nested PCR, 253 (93.35%) amplicons (848 bp) were obtained from the DNA of 271 samples as visualized after SYBR®Safe DNA Gel Stain (Invitrogen) staining of the gel. After ClustalW alignment and Jalview analysis of the retrieved sequences, this study found one synonymous and one non-synonymous mutation in Bangladeshi clinical isolates (Table 1). Geospatial mapping of the districts from which samples were obtained are depicted (Figure 1). The mapping demonstrates that the non-synonymous mutations are in the K13 protein locus of *P. falciparum* samples (n = 2) from the Matiranga UHC of Khagrachari district. Two samples from unrelated individuals living 15 km apart contained the same A578S mutation in the K13 propeller gene. Genotyping of the isolates using *msp1* and *msp2* demonstrated the isolates were genetically distinct (data not shown). Matiranga UHC is close to the border with India.

**Table 1:** **Distribution of samples and non-synonymous mutations by District and Upazila Health Complex (UHC) in Bangladesh**

District name	Upazila Health Complex (UHC)	Samples (N)	K13 Nested PCR and sequencing N (%)	Non-synonymous Mutation N (%)
Bandarban	Lama UHC	14	149(100)	0(0.0)
Cox’s Bazar	Ramu and Ukhia UHC	74	69(93.2)	0(0.0)
Netrokona	Durgapur and Kalmakanda UHC	10	9(90)	0(0.0)
Mymensingh	Haluaghat UHC	2	2(100)	0(0.0)
Khagrachari	Matiranga UHC	154	144(93.5)	2*(1.29)
Rangamati	Rajasthali UHC	6	5(66.7)	0(0.0)
Moulvibazar	Sreemangal and Kamalganj UHC	11	11(100)	0(0.0)
	Total	271	254(93.7)	2(0.78)

*The mutation A578S was identified in two unrelated individuals with genetically distinct isolates.

**Figure 2:**
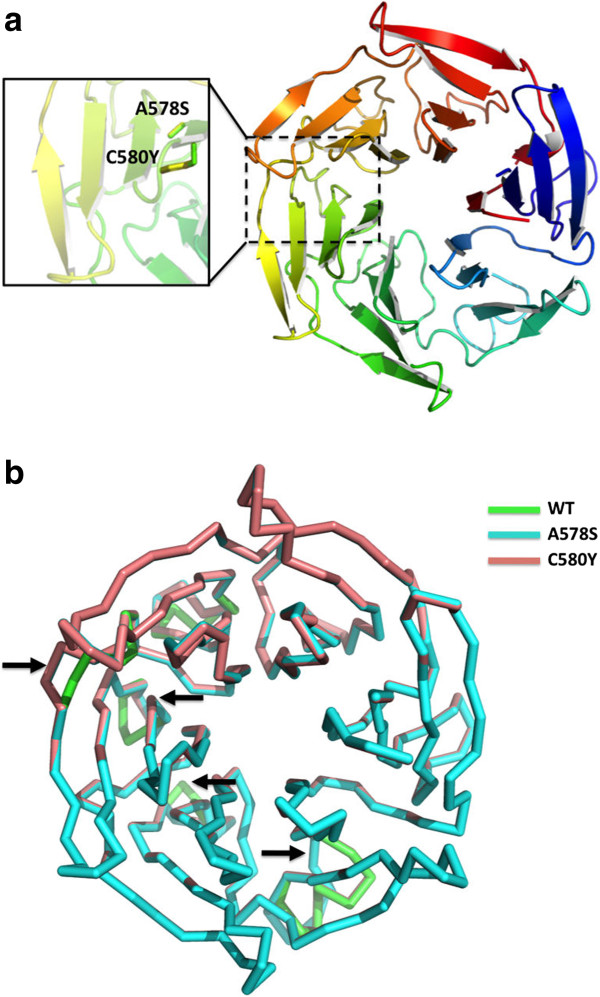
**Predicted model of the** ***P. falciparum*** **K13 propeller protein. a)** Location of the mutation sites relative to the overall model of the protein. The beta-propeller domain of the btb-kelch protein Krp1 (PDB ID: 2WOZ) was used as the modeling template. **b)** Superimposition of the homology models of the wild type and mutated proteins. Areas where the individual colors of the mutants are illustrated display regions where the mutation affects the predicted fold. These areas are indicated by the black block arrows.

Computational modeling was performed using the beta-propeller domain of the btb-kelch protein krp1 (PDB ID: 2WOZ). The resulting model (Figure 2a) resembled closely the topology and statistics of the predicted model by Ariey [5] as assessed by sequence alignment to the coordinates of the template and prediction quality. The model predicts a 6-bladed propeller structure with homology to previously characterized kelch domain proteins [23, 24]. Mutational sensitivity prediction by the PHYRE2 Investigator suggests that the A578S mutation observed in this study is likely to disrupt the *P. falciparum* K13 propeller protein function. K13-propeller protein mutations are reported to alter the biological function of the protein by modification of surface charges that disrupt interactions with other proteins. Superimposition of the homology models of the wild type and mutated proteins show qualitatively that A578S mutant (in addition to the known mutant C580Y seen in South East Asia) is predicted to cause structural changes in the overall topology of the protein (Figure 2b). Due to these changes, the function of the protein with these mutations is quite likely disrupted. Indeed, these modeling predictions are consistent with the findings of the study by Ashley [25] who report that mutations of the K13 propeller protein after amino acid position 440 were associated with a mean increase in the parasite clearance half-life of 116% (95% CI, 103 to 131; P < 0.001) [26].

## Discussion

Although the history of ACT use in Bangladesh dates to only 2004, the country lies adjacent to countries where resistance to ACT is emerging. Parasites from South East Asian countries appear to have a propensity to become multi-drug resistant [3]. Researchers have observed that mutations such as M476I in the K13 propeller gene are linked to artemisinin resistance *in vitro* using the ring stage assay (RSA) developed specifically to test artemisinin activity in the test tube [5]. Clinical studies have also confirmed 17 mutations in the K13 propeller gene were associated with delayed parasite clearance in Cambodia [5], contrasting data showing high efficacy of ACT in Bangladesh [10–12] in the Bandarban district. This study has collected blood specimens from different parts of the country and evaluated the presence of K13 propeller mutations. Two (0.78%) specimens from unrelated individuals appear to contain structure-altering mutations (A578S) in the K13 propeller gene from genetically distinct isolates. These data suggest that mutations in K13 arise *de novo* rather through clonal expansion. The Matiranga UHC in the district of Khagrachari is in the tri-border area with Myanmar and India. There is no report of artemisinin resistance from India so far. While ACT remains efficacious and these mutations are rare based on this surveillance, ACT usage will impose positive selection on the resistant parasite. It has been suggested that the high diversity of vector species in Bangladesh [27, 28] can contribute to the spread of these mutated strains of *P. falciparum.* Moreover, a very low rate of transmission can result in inadequate immunity to clear the parasite that might have survived after ACT treatment, thereby increasing the selection pressure on resistant clones [26]. Monitoring of possible resistant parasite clones is of paramount importance. A limitation of this study is the lack of clinical efficacy data. Also no other genetic markers of resistance were evaluated.

Protein modelling data suggest that A578S can alter the function of the K13 propeller protein. A578S is very close to the C580Y mutation, the major mutation observed in the K13 propeller protein of the Cambodian isolates [5]. The tracking resistance to artemisinin collaboration (TRAC) group also found the A578S mutation in one specimen within their study areas but this mutation was not associated with increased parasite clearance half-life [25]. Importantly, mutations such as A578S lie after the position 400 and alter the structure of K13 significantly and thus may have an effect on artemisinin activity. Crude measures like parasite clearance time are multi-factorial phenotypes influenced by pharmacokinetics, immunity, and patient adherence, and thus may not immediately correlate with important mutations without a large sample size. Ramu UHC in Cox’s Bazar district is one of the TRAC study sites where slow clearing parasites were observed without any mutation on K13 propeller gene which again speaks to the multi-factorial nature of this phenotype. Similarly, clinical specimens collected from Ramu in the study presented here did not demonstrate mutations in the K13 gene. Takala-Harrison [29] recently demonstrated that the vast majority of isolates in their collection were wild type at the K13 locus with A578S not present.

## Conclusion

Up-to-date information on whether artemisinin resistance has already disseminated or independently emerged beyond the Cambodia-Thailand region is a significant issue. The WHO, with the help numerous collaborators, has declared the global plan for artemisinin resistance containment [30]. If resistance were limited to a small, well-defined area, then elimination of drug-resistant malaria from these regions would be possible [31]. Unfortunately, resistance is not limited to the Thai-Cambodian border, it has already spread to the Vietnam, Laos and Myanmar border [25]. Our data demonstrate that mutations strongly linked to artemisinin resistance are not present at this time in Bangladesh.
